# The Inventory of Quality in Early Intervention Centres for Service Providers: Preliminary Validating Study in a Spanish Sample

**DOI:** 10.3390/ijerph17072581

**Published:** 2020-04-09

**Authors:** Inmaculada-Concepción Jemes-Campaña, Rita-Pilar Romero-Galisteo, María-Teresa Labajos-Manzanares, Pablo Gálvez-Ruiz, Noelia Moreno-Morales

**Affiliations:** 1 Doctoral School, University of Málaga, 29071 Málaga, Spain; ijemes@uma.es; 2 Department of Physiotherapy, Faculty of Health Sciences, University of Málaga, 29071 Málaga, Spainnmm@uma.es (N.M.-M.); 3 Valencian International University, 46002 Valencia, Spain; pgalvez@universidadviu.com

**Keywords:** service quality, early intervention, factorial structure, validity, service providers

## Abstract

Evaluating the service quality in early intervention (EI) from the perspective of professionals with knowledge in this area is essential for the improvement of EI centres. In this study, we aimed to test the reliability and validity of the adapted Inventory of Quality in Early Intervention Centres-P (IQEIC-P) in a sample of professionals who worked in EI centres. Three hundred and twenty-four professionals of 85 EI Spanish centres were recruited for this research. Various psychometric analyses were used to evaluate the factorial structure, the internal consistency, factorial validity and construct validity. A 5-dimension structure was obtained in the exploratory factor analysis (EFA). The results showed an adequate internal consistency (Cronbach’s alpha values between 0.71 and 0.83, and composite reliability (CR) values higher than 0.70), as well as satisfactory convergent and discriminant validity (average variance extracted (AVE) values above 0.50). In the confirmatory factor analysis, good model fit indicators were observed. The IQEIC-P showed adequate psychometric properties, demonstrating to be a valid instrument for the evaluation of service quality in EI centres from the perspective of professionals. The benefits will influence the professionals themselves, and they will have a positive and direct impact on the families that are attended to in these centres.

## 1. Introduction

One of the objectives of early intervention (EI) is to respond to the temporary or permanent needs of children with developmental disorders or at risk of developing such [1]. EI acts in the first years of a child’s life, which are decisive for his/her neurodevelopment [2,3].

EI is undergoing an important transformation process since a few years ago [4,5,6], where offering a quality service has become a topic of interest. In this sense, knowing the key elements that determine a positive evaluation of service quality [7], as well as knowing how to define and measure such a construct, is currently fundamental. There is a lack of consensus on measuring EI service quality, even after decades of studies [8], due to the fact that these dimensions cannot be generalised to any service or geographical context [9]. Thus, it is necessary to adapt the tools, and this creates the need to develop specific scales that use dimensions with a more direct relationship depending on the context [10].

EI services in Spain are organised differently in each of the autonomous communities into which the country is divided. Each of these has the authority to organise the services that it provides in a different way [11]. The ministries with responsibility for EI are Health, Social Services and Education, which must coordinate with each other to ensure quality services based on prevention, early identification and intervention [1]. EI programs, most of which are subsidised by the government, are organised in Early Intervention Centres (EIC) [12].

According to the White Paper on Early Intervention [1], Early Intervention Centres (EIC) are defined as specialised centres, with suitable infrastructure and multi-disciplinary personnel, responsible for providing integral attention to the underage and to their families and environment. This organisational structure is the most extended in the Spanish territory. In the specific case of Andalusia (Southern Spain), it is legislated according to Decree 85/2016, of 26 April, which regulates the integral intervention of Early Childhood Care [13], although in other countries the welfare model may be different from the one applied in Spain [14].

Given the need to develop tools contextualised for the territory in which they will be used [10], Romero-Galisteo et al. [15] designed the “Inventory of Quality in Early Intervention Centres” (IQEIC) to evaluate the quality perceived by the families attended to in EI centres. 

This tool was constructed from the Questionnaire of Perceived Quality in Early Intervention Physiotherapy [16] and the Satisfaction of the Client Family Questionnaire [17]. IQEIC was a scale composed of four dimensions (centre facilities, treatment rooms and material, qualified staff and technical or specific information) and 26 items. The response format for all items is a 5-point Likert scale rated from 1 (strongly disagree) to 5 (strongly agree). 

The questionnaire has adequate psychometric properties, with a good fit of the indices considered in the evaluation of the model, as well as satisfactory reliability (composite reliability) and validity (convergent and discriminant). Previous versions of IQEIC have demonstrated the validity and reliability of the questionnaire [15,18,19]. To the best of our knowledge, after carrying out a literature review, the scale proposed by Romero-Galisteo et al. [15] is the only one validated in this study context [20].

In the evaluation of quality, the perspective of the patient has increased considerably [21], and, in the case of EI, the family is a fundamental aspect in the analysis of service quality [18,22,23]. However, the academic literature suggests that evaluation should not be exclusively focused on the family [24], as it is necessary to analyse the perspective of professionals with knowledge in this area.

Service providers must be involved in the evaluation of the quality of the services they provide, as this partially determines the success of their management [25]. This author highlights the relevance of comparing the perceptions of the clients with those of professionals. In this line, several authors [26,27] state that it is essential to know the opinion of the professionals since it directly affects the design, dedication, and results of the service provided. Therefore, it is important to consider the perception of the professionals who compose the multidisciplinary teams in EI centres [1,28] for the design of specific measuring tools [29]. However, the perspective of professionals has been studied only as another dimension of service quality [28].

In view of this situation, and due to the lack of tools for the evaluation of service quality from the perspective of the professionals, the relevance of the present study emerges from the need detected in this specific context [20].

Evaluating and comparing the perspectives of families and professionals will allow advancement in the process of continuous improvement that EI centres (EI) are undergoing. To this end, it is necessary to develop valid and reliable tools that consider the context in which they were created and in which they will be used.

Thus, the main objective of this study was to adapt a specific instrument to measure the service quality perceived by the professionals of EI centres. In the literature, exploratory and confirmatory factor analyses are used to test the construct validity of scales. Therefore, despite the previously known structure, both analyses were used in this study, since the adaptation of the items can give rise to new factorial structures. In this way, with the aim of exploring the dimensionality of the tool, we conducted an exploratory factor analysis with the relevant tests (Kaiser–Meyer–Olkin (KMO) and Bartlett’s test of sphericity) and a confirmatory factor analysis to verify the obtained structure by adding the complementary measures of reliability and validity (composite reliability and average variance extracted) [30,31].

## 2. Materials and Methods

### 2.1. Participants 

Three hundred and twenty-four professionals from 85 EI centres of the region of Andalusia were recruited to participate in this study. The respondents were 35.7 years old on average (SD = 8.83). Regarding gender, the sample included 86.4% female and 12.7% male respondents (missing data 0.9%). With respect to the profession of the participants, eighty-eight were physiotherapists, eighty-five psychologists, seventy-five speech therapists, sixteen occupational therapists and forty-six had other professions. With regard to years of experience in early intervention, one hundred and forty participants had 0–5 years of experience, seventy-six had 6–10 years, seventy-two had 11–20 years and nineteen had more than 20 years (Table 1).

The questionnaires were sent to these centres via ordinary mail and identified with a numerical code. Authorisation was requested from the different centres through a letter explaining the purposes of the investigation and the procedure to be carried out. The participants were informed of the purpose of the study, and informed consent was requested from them to participate, in accordance with the Declaration of Helsinki, as well as the scientific nature of the study and the anonymity and confidentiality of the responses. The data collection was carried out during the months of May and June of 2018. Permission to undertake this study was granted by the Research Ethics Committee at Malaga University (code 22-2018-H). The exclusion criteria established were the following: informed consent not completed and not having enough knowledge for correctly understanding and expressing the Spanish language.

### 2.2. Measures

The Inventory of Quality in Early Intervention Centres (IQEIC) [15] was adapted to determine the valuation of ECI service quality from the perspective of professionals who work in EI centres, obtaining an adequate fit to the data (χ^2^;/gl = 2.53, CFI = 0.94, TLI = 0.92, IFI = 0.94, RMSEA = 0.059). This questionnaire is originally composed of 26 items distributed in four dimensions following the structure of the model proposed in the study of Romero-Galisteo et al. [15]. The first dimension is called “centre facilities” (CF), and it comprises the items related to accessibility, location and adequacy of the facilities; the second dimension would be “treatment room and material” (TRM), which groups the items related to the state of both the treatment rooms and the materials used in them; the third dimension, called “qualified staff” (QS), evaluates the professionals; and the last dimension, called “technical or specific information” (TSI), is related to the more specific and detailed information given to the families.

After adapting the items, the next step was the determination of the content validity by three experts in EI with more than 10 years of experience, who read and confirmed the correct understanding of all the items with no need to remove any of them, suggesting the punctual adaptation of some words in several items (e.g., “*the activities proposed for users to work outside the centre are feasible*” instead of “*the activities proposed for users to work on at home are feasible*”; “*the information it gives about the user is clear*” instead of “*the information received about the user is clear*”). A Likert-type scale was used, ranging from “1” = *completely disagree* to “5” *completely agree*. In addition, the questionnaire included a first section with sociodemographic questions, aimed at identifying the characteristics of the participants in the study.

### 2.3. Statistical Analyses

Various psychometric analyses were used in this study. The sample was randomly divided into 2 parts to carry out the evaluation of the psychometric properties and construct validity. The first part represented approximately 35% of the sample (*n* = 112), which was enough for the exploratory factor analysis (EFA) calculation [32], utilizing principal component extraction and Oblimin oblique rotation as a method of factor extraction. Beforehand, Kaiser–Meyer–Olkin (KMO) and Bartlett sphericity tests were conducted to determine whether the items were significantly correlated and shared sufficient variance to justify factor extraction [33]. The internal consistency of each scale was assessed using Cronbach’s alpha [34,35] and by examining the corrected item–total correlations between the individual items [31,36]. To determine the relationships among variables, the inter-correlation of the items was evaluated with Pearson’s correlations.

The second part represented approximately 65% (*n* = 211) of the sample and was used for the confirmatory factor analysis (CFA) to test the fit of the measurement model using the maximum likelihood estimation. Composite subscale scores were then created by calculating the mean of all the items in a given subscale. With regard to goodness-of-fit indices, we used χ^2^ and its differences regarding the degrees of freedom (χ^2^/df), comparative fit index (CFI), Tucker Lewis index (TLI), incremental fit index (IFI) and root mean square error of approximation (RMSEA). We considered the fit indicators to be adequate when χ^2^/df was below 3 [37], CFI, TLI and IFI were equal to or above 0.90, and RMSEA was below 0.08 [31,38]. The statistical analyses were conducted using SPSS and AMOS 21.0 (SPSS Inc, Chicago, 127 IL, USA).

In addition, the internal consistency of the constructs was measured through composite reliability [31], and convergent validity was evaluated through the average variance extracted (AVE), whilst discriminant validity was also established when the AVE for each latent construct exceeded the squared correlations between that construct and any other [39].

## 3. Results

No missing data were found in the answers, and the different items obtained a high average score, with those related to the information about people who work in EI centres being the ones with the highest score. Table 2 presents the descriptive statistics (i.e., mean, standard deviation [SD]) and internal consistency of the different dimensions of the questionnaire, and correlations between dimensions.

The relevance of the EFA showed a satisfactory KMO sampling adequacy index (0.902) and significance according to Bartlett’s test of sphericity [χ^2^(325) = 3758.77; *p* < 0.001]. The results explain 58.32% of the total variance explained, indicating that the scales had satisfactory factor structures [33] showing sample adequacy and suitability for completing a factorial analysis. The EFA showed the existence of five dimensions, as it identified a new dimension (intervention), composed of two items (5.61% of the total variance). Factor loadings above 0.40 were obtained, which is the saturation criterion to consider that an element is an indicator of the factor [40], with the exception of items 13 (“the specialised staff are easily identifiable”; λ = 0.256) and 18 (“users value the contributions and initiatives of the qualified staff”; λ = 0.236). All the items presented commonalities above 0.50 [41].

The questionnaire had optimal internal consistency reliability (α = 0.90). However, after deleting items 13 and 18, very good reliability levels were obtained, between 0.70 and 0.83. The correlation coefficient of each item score and the total score (HIc index, Table 3) of IQEIC-P was between 0.266 and 0.745; in all cases, the values were greater than 0.25 [42]. The correlations between the dimensions were significant and moderate, between 0.37 and 0.71 (Table 2).

According to the descriptive analyses, the dimension that was best valued by the participants was “qualified staff”. On the other hand, the dimension with the lowest score was “treatment room and material”, which was the only one with a mean value below 4 (Table 2).

Regarding the items, the professionals evaluated accessibility and the relationship with the users in a positive manner (items 16,17). In contrast, they gave a lower valuation in those items related to the materials available to carry out the intervention (item 10) (Table 3).

**Figure 1:**
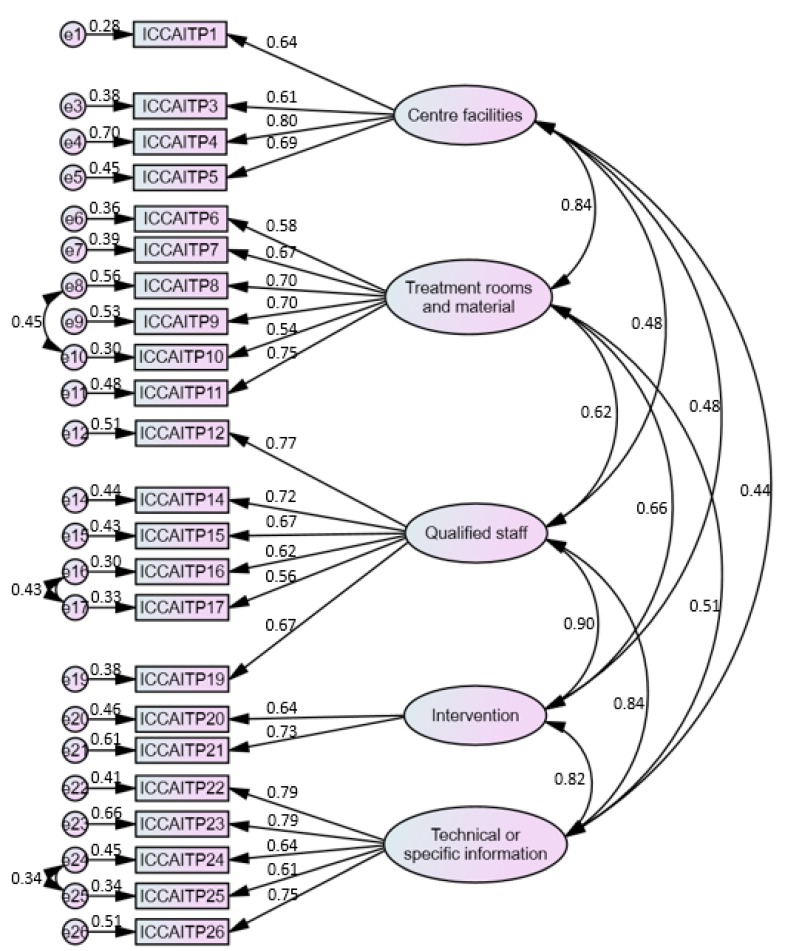
Confirmatory factor analysis estimates of the 23-item questionnaire.

The values pertaining to the original confirmatory factor analysis (CFA) model with 26 items showed poor results. Although the value of χ^2^/df was good (2.39), those of CFI (0.834), TLI (0.813), IFI (0.836) and RMSEA (0.085) were below the recommended level, and these results indicated that the model should be adjusted. The items showed a factor loading greater than the conservative threshold of 0.60 (R^2^ ≥ 0.30) [33], indicating adequate individual reliability, except for items 2 (λ = 0.311; “it is easy to get to the centre using public transportation”), 13 (λ = 0.417) and 18 (λ = 0.490), which were consequently removed. In addition, according to the modification indices (MI) and theoretical knowledge, we established covariant relations between e8 and e10 (MI = 47.23), e16 and e17 (MI = 41.74) and e24 and e25 (MI = 21.03). The result was a scale composed of 23 items with an acceptable fit to the data in CFA [χ^2^/df = 1.68; CFI = 0.934; TLI = 0.923; IFI = 0.935; RMSEA = 0.057] (Figure 1).

The convergent validity is demonstrated in two ways. First, the factor loadings were significant and greater than 0.5 [43,44], and, second, the average variance extracted (AVE) for each construct was higher than 0.5 [31]. The composite reliability (CR) of the scale is also demonstrated, as the indices of each of the dimensions obtained were higher than the threshold of 0.7 [43,44]. Lastly, the discriminant validity of the measurements was accepted, given the square correlations between each dimension and any others that were lower than the AVE values for each dimension [31] (Table 4).

## 4. Discussion

EI and the services provided to children with disabilities have gained interest in recent studies [18,24,44,45]. However, few researchers have approached the service quality through the use of validated tools to evaluate early childhood intervention centres from the perspective of professionals who work in these institutions. Therefore, this study aimed to determine the psychometric properties of an instrument adapted to this specific context, with the aim of filling the gap in the literature regarding reliable and valid instruments that allow measuring the quality perceived by the population of EI professionals.

Thus, the instrument presented is an adaptation of the IQEIC questionnaire [15]. Despite having all the original items after the content validation, the methodological criteria applied led to the removal of three items, which did not show adequate results. The dimensions that comprised the tangible aspects, specifically CR and TFM, obtained the lowest scores, whereas the dimensions related to the intervention of professionals, such as QS, I and TSI, showed higher valuations. These results show similarities with those obtained by Horridge et al. [46], where the valuations of the professionals were lower in aspects related to the facilities and treatment rooms. These results could be due to the cutbacks to early childhood intervention centres and treatment equipment [47]. Moreover, they reported on issues related to the waiting time, as well as on the reduction in the time allocated to the treatment of children, which had a negative influence on the perception toward service quality. It is worth highlighting the score difference regarding the items of dimension I; its results were lower than those of the present study. This could be related to the negative connotations of the term “economic cutbacks”, encouraging the participants to give lower scores, and even possibly influencing the authors’ interpretation of the results.

One of the important aspects of measuring the perceived quality is that it allows the identification of criteria that create opportunities for improvement. In fact, the results of measuring the current situation could provide the necessary motivation for quality improvement [48], becoming important for professionals who provide these services, especially for those in positions of responsibility, for adequate decisions making. In this sense, the benefits do not only influence the professionals themselves but will also have a positive and direct impact on the families that are attended to in these centres.

In other countries, several studies have evaluated the service quality from the perspective of the professionals [4,27]. Their results show significant differences between the occupations or job positions of the participants. Similarly, the instrument presented in this study considered the participation of the multi-disciplinary teams that work in early childhood intervention centres, that is, physiotherapists, speech therapists, psychologists and occupational therapists. Thus, a broader perception was obtained, representing an advantage with respect to other tools which base the perception of the service on specific professionals, such as physiotherapy [14,16,49]. 

Some of the tools used nowadays to evaluate the service quality from the perspective of the professionals are based on other tools that were previously designed for the families. An example of these is the Measure of Processes of Care for Service Providers (MPOC-SP) [50], whose structure is similar to that of MPOC-56 [51], which is the version for family members. Studies in which both scales were used [51] highlight the need to develop the questionnaire for the professionals with parallel dimensions with respect to the version for the families, with the aim of facilitating the comparison of both perspectives. 

Aytch et al. (1999) [52], among other authors, consider that using the same tool for different populations is inadvisable. In fact, they question the development of questionnaires based on the perception of a single population group since families, service providers and researchers can disagree on the aspects to be evaluated. Therefore, for the design of the tool presented in this work, we used both the latent variables proposed by Romero-Galisteo et al. [15] and the contributions of experts in EI with more than 10 years of experience.

Although the results obtained in previous studies [50,53,54] show differences between the valuation of “providing general information” and that of “technical or specific information”, whose content is similar, higher values were obtained in “treating people respectfully” and “qualified staff”.

Other studies compare the results obtained between professionals and families, showing in some cases similar values [55], and opposing opinions in other cases [56], which also demonstrates the need to analyse both perspectives [25].

Moreover, the dimensions used allow approaching the quality construct in a wider way compared to other studies that approach the model focused on the family [11,14,50,55] without considering the tangible aspects of the service (facilities, waiting rooms, state and quality of the material, etc.). Furthermore, the obtained results show the existence of a new dimension with respect to the original version of the tool, in this case, called “intervention”. Although this dimension was difficult to value for the families, it is of great relevance for professionals, which confirms that the dimensions cannot be generalised to different contexts [9]. This is in line with the methodological limitations obtained by other studies that used the same instrument in different scopes [57,58,59]. Nevertheless, authors, such as McWilliam et al. [60], evaluated the “intervention planning” from the perspective of the families.

Lastly, the purpose of designing this type of tool is to develop and contribute to the implementation of a process of continuous improvement, which is necessary in the service quality culture of early childhood intervention centres, facilitating their evaluation and the evaluation of their services, and allowing the implementation of improvements both in the short and long term [48,61].

However, the present study has some limitations. One of them is related to the sample recruitment. Since it was purposive sampling, the results cannot be generalised to other contexts, such as hospitals, private pediatric clinics, intervention exclusively in natural environments, or other geographic regions. Therefore, we consider it necessary to verify the factor structure in other contexts where the administrative organisation of EI services is different from that of early childhood intervention centres [62]; thus, studies which demonstrate the factor invariance of the tool could be very useful. In this line, future studies must compare the different perspectives, of both users and professionals, with the aim of advancing in the process of continuous improvement. On the other hand, analysing the influence of certain sociodemographic factors, such as time worked, on the perception toward service quality, would be another research line that would undoubtedly contribute with interesting data to this research field.

Another possible limitation is that a self-completion questionnaire is presented; thus, it is necessary to interpret the results cautiously [63]. However, self-evaluation is recognised as a useful tool that provides relevant information [64,65].

## 5. Conclusions

The obtained results allow asserting that the IQEIC-P instrument provides adequate psychometric properties for the evaluation of service quality in early childhood intervention centres from the perspective of EI professionals. The five dimensions that make up this tool provide relevant information both for the multi-disciplinary teams that work in the centres and for their managers. The evaluation of service quality will allow identifying areas for improvement, which requires future studies to delve into this construct in different contexts.

## Figures and Tables

**Table 1:** Sociodemographic characteristics of the participants.

Variables	*n*	%	Missing (%)
*Gender*			3 (0.9)
Male	41	12.7	
Female	280	86.4	
*Age (years)*			15 (4.6)
20–30	124	38.3	
31–40	115	35.5	
41–50	51	15.7	
>50	19	5.9	
*Profession*			14 (4.3)
Psychology	85	26.2	
Physiotherapy	88	27.2	
Speech Therapy	75	23.2	
Occupational Therapist	16	4.9	
Others	46	14.2	
*Experience (months)*			17 (5.2)
0–5	140	43.2	
6–10	76	23.5	
11–20	72	22.2	
>20	19	5.9	

**Table 2:** Internal consistency, descriptive analysis and correlations between dimensions.

Dimensions	α	M (SD)	CF	TRM	QS	I	TSI
Centre Facilities (CF)	0.71	4.08 (0.71)	1				
Treatment Room and Material (TRM)	0.83	3.96 (0.70)	0.65 **	1			
Qualified Staff (QS)	0.81	4.51 (0.48)	0.38 **	0.46 **	1		
Intervention (I)	0.70	4.35 (0.66)	0.35 **	0.47 **	0.71 **	1	
Technical or Specific Information (TSI)	0.81	4.43 (0.54)	0.37 **	0.38 **	0.66 **	0.61 **	1

Note. α = Cronbach alpha coefficient; M = Mean; SD = Standard Deviation; ** = *p* < 0.005.

**Table 3:** Mean, standard deviation, factor loadings in exploratory factor analysis (EFA), confirmatory factor analysis (CFA) and homogeneity index corrected (HIc).

Dimensions and Items	M (SD)	EFA	CFA	Hic
*Centre Facilities*				
1. The centre is well located geographically	4.27 (0.95)	0.825	0.643	0.266
3. The cleaning of the centre is adequate	4.05 (1.23)	0.652	0.610	0.471
4. The centre facilities are comfortable	4.03 (0.95)	0.849	0.803	0.636
5. The facilities have the correct signalling	3.99 (1.06)	0.633	0.694	0.544
*Treatment room and material*				
6. The number of treatment rooms is enough	3.72 (1.17)	0.724	0.584	0.514
7. The treatment rooms are large enough	4.10 (1.03)	0.729	0.679	0.570
8. The materials that are used in the centre are suitable	3.92 (0.92)	0.826	0.709	0.745
9. The materials are in good condition for use	4.05 (0.85)	0.763	0.707	0.654
10. The working material used in the centre is sufficient	3.58 (1.03)	0.802	0.545	0.612
11. The materials that are used in the treatment rooms are safe	4.44 (0.71)	0.644	0.751	0.566
*Qualified staff*				
12. The attention paid to users at the centre is adequate	4.51 (0.62)	0.600	0.771	0.628
14. The attention paid to users at the centre is sufficient	4.14 (0.79)	0.540	0.729	0.568
15. The qualified staff have the necessary knowledge	4.51 (0.69)	0.634	0.671	0.576
16. The qualified staff are accessible	4.72 (0.56)	0.768	0.621	0.613
17. The specialised staff have a close relationship with the users	4.79 (0.48)	0.755	0.565	0.563
19. The qualified staff are coordinated with one another to improve and complete the user’s service	4.43 (0.81)	0.760	0.667	0.542
*Intervention*				
20. The specialised staff of the centre are coordinated with other services to improve and complete the user’s service	4.37 (0.80)	0.749	0.647	0.543
21. The activities carried out with the user seem appropriate	4.34 (0.70)	0.684	0.739	0.543
*Technical or specific information*				
22. The activities proposed to work with the user outside of the centre are feasible	4.00 (0.96)	0.679	0.792	0.672
23. The information received at the beginning of the treatment is consistent with the tasks performed subsequently	4.46 (0.69)	0.679	0.792	0.672
24. The families are given instructions/programs to work with the user outside of the centre	4.56 (0.66)	0.779	0.640	0.651
25. The centre provides a report (oral/written) about the progress of the user	4.58 (0.67)	0.809	0.618	0.574
26. The information given about the user is clear	4.59 (0.57)	0.765	0.756	0.660

Note. M = Mean; SD = Standard deviation; EFA = Exploratory factor analysis; CFA = Confirmatory factor analysis; Hic = Homogeneity index corrected.

**Table 4:** Composite reliability, average variance extracted and discriminant validity.

Dimensions	CR	AVE	CF	TRM	QS	I	TSI
Centre Facilities (CF)	0.80	0.50	1				
Treatment Room and Material (TRM)	0.86	0.51	0.438	1			
Qualified Staff (QS)	0.85	0.51	0.228	0.497	1		
Intervention (I)	0.76	0.61	0.234	0.433	0.355	1	
Technical or Specific Information (TSI)	0.83	0.52	0.199	0.262	0.475	0.404	1

Note. CR = Composite Reliability; AVE = Average Variance Extracted.
